# Photocatalytic Degradation of Pharmaceutical Residues from Water and Sewage Effluent Using Different TiO_2_ Nanomaterials

**DOI:** 10.3390/nano14020135

**Published:** 2024-01-06

**Authors:** Amir Hossein Navidpour, Mohammad Boshir Ahmed, John L. Zhou

**Affiliations:** 1 Centre for Green Technology, School of Civil and Environmental Engineering, University of Technology Sydney, Ultimo, NSW 2007, Australia; amirhossein.navidpour@student.uts.edu.au; 2 Institute for Sustainability, Energy and Resources (ISER), School of Chemical Engineering, The University of Adelaide, North Terrace, SA 5005, Australia; mohammadboshir.ahmed@adelaide.edu.au

**Keywords:** colloids, pharmaceuticals, photocatalysis, sewage effluent, titanium dioxide

## Abstract

Pharmaceuticals are widely used and often discharged without metabolism into the aquatic systems. The photocatalytic degradation of pharmaceutical compounds propranolol, mebeverine, and carbamazepine was studied using different titanium dioxide nanostructures suspended in water under UV and UV-visible irradiation. Among three different photocatalysts, the degradation was most effective by using Degussa P25 TiO_2_, followed by Hombikat UV100 and Aldrich TiO_2_. The photocatalytic performance was dependent on photocatalyst dosage, with an optimum concentration of 150 mg L^−1^. The natural aquatic colloids were shown to enhance the extent of photocatalysis, and the effect was correlated with their aromatic carbon content. In addition, the photocatalysis of pharmaceuticals was enhanced by the presence of nitrate, but inhibited by the presence of 2-propanol, indicating the importance of hydroxyl radicals. Under optimum conditions, the pharmaceuticals were rapidly degraded, with a half-life of 1.9 min, 2.1 min, and 3.2 min for propranolol, mebeverine, and carbamazepine, respectively. In treating sewage effluent samples, the photocatalytic rate constants for propranolol (0.28 min^−1^), mebeverine (0.21 min^−1^), and carbamazepine (0.15 min^−1^) were similar to those in water samples, demonstrating the potential of photocatalysis as a clean technology for the effective removal of pharmaceuticals from sewage effluent.

## 1. Introduction

Of current concern are pharmaceutical compounds which are widely used in human and veterinary medicines, and eventually find their way into the natural environment with potential long-term adverse effects [1,2]. Many pharmaceuticals are poorly absorbed by the human body and are mostly excreted with feces and urine without significant metabolism [2,3,4]. In addition, many different pharmaceuticals do not possess chromophores and are highly persistent towards hydrolysis, adsorption, biodegradation, and abiotic degradation [3]. As a result, such pharmaceutical compounds are not effectively removed during sewage treatment [2,5], as sewage treatment works (STWs) are still geared towards traditional pollutants such as heavy metals, chemical oxygen demand (COD), and 5-day biochemical oxygen demand (BOD_5_). Furthermore, sewage effluents and hospital effluents are important point sources of pharmaceutical residues in surface water and groundwater [5,6,7]. The potential impact of pharmaceutical residues on aquatic organisms and potentially humans through endocrine-disrupting effects or pathogenic bacteria developing antibiotic resistance [8,9] is a global major concern.

Advanced wastewater treatment is therefore needed to enhance the removal of pharmaceuticals from sewage effluent. A full-scale commercial granular activated carbon (GAC) plant has been operating in a major STW and proven effective in removing many pharmaceutical compounds [7]. However, the associated high capital and running cost for GAC plants could prohibit their wide applications, particularly in the developing world. Other methods such as advanced oxidation processes (AOPs) have been widely studied, which rely on the production of very reactive oxygen species (ROS) such as hydroxyl radicals (^•^OH) and singlet oxygen (^1^O_2_) for the oxidation of organic pollutants such as pharmaceuticals [10]. 

Among the different AOPs, photocatalysis using semiconductors such as TiO_2_ represents a clean technology which has been studied and is becoming popular for the degradation of pharmaceuticals [8,11]. Various metal oxides such as Ga_2_O_3_ [12], ZnO [13,14], TiO_2_ [15,16,17], and In_2_O_3_ [18], polymeric materials such as g-C_3_N_4_ [19,20,21,22], and spinel structures such as CuFe_2_O_4_ [23] and ZnFe_2_O_4_ [24] have been used in photocatalysis technology. Notably, TiO_2_ is generally regarded as the benchmark photocatalyst [25]. It has the advantages of abundancy, structural diversity, and high chemical stability [26]. Furthermore, it has found applications in sodium-ion batteries [27]. In photocatalysis, the irradiation of light in the presence of a photocatalyst could lead to the generation of charge carriers as follows [28]:

(1) $$Photocatalyst+h\upsilon \to e_{CB}^{-}+h_{VB}^{+}$$

The photo-excited e^−^ and h^+^ species then generate extremely ROS such as hydroxyl (^•^OH) and superoxide radicals (O_2_^•−^) at the semiconductor surface that cause the oxidation of pharmaceutical molecules [29]. In addition, sulfate radicals (SO_4_^•−^) are increasingly been used for environmental photocatalysis applications [30]. The photocatalytic degradation of pharmaceuticals such as diclofenac, paracetamol, ibuprofen, oxolinic acid, amoxicillin, and memantine has been previously examined [3,31,32,33,34]. However, most studies tend to be conducted using very high pharmaceutical concentrations (e.g., mg L^−1^), which are typically several orders of magnitude higher than those found in sewage effluents or natural waters (ng L^−1^–μg L^−1^ range). In addition, the process of indirect photolysis can be influenced by many factors, including the properties of the photocatalyst, the physicochemical properties of the target compounds, and environmental conditions [5,35].

The effect of dissolved organic matter (DOM) on the photochemical transformation of organic contaminants has been widely studied, due to the ubiquitous occurrence of DOM in natural water and wastewater [10,36]. It has been reported that DOM can become excited, under solar irradiation, to singlet state before being rapidly elevated to the excited triplet states (^3^DOM*) [37]. In particular, dissolved black carbon was reported to be six times more effective than humic substances in photosensitizing the photo-transformation of 17β-estradiol in water [10]. However, there are also reports that DOM inhibited the photochemical processes, which was suggested to be due to the DOM absorption of photons and thus the quenching of radicals [35,38]. Therefore, further investigation of the DOM effect on pharmaceutical photodegradation is warranted.

This work aimed to examine the photocatalysis of three pharmaceuticals (propranolol, mebeverine, and carbamazepine) under realistic environmental concentrations. They were chosen based on their high-risk characterization ratio, quantity used, and wide occurrence. The reaction kinetics, environmental factors affecting the photodegradation process, and the potential role of radicals were investigated. The application of photocatalysis to the treatment of secondary sewage effluents was also assessed as a potential low-cost green technology.

## 2. Materials and Methods

### 2.1. Material and Reagents

The pharmaceutical compounds propranolol, mebeverine, and carbamazepine were obtained from Sigma-Aldrich, Sydney, Australia. The internal standards (diuron-d_6_ and ^13^C-phenacetin) were supplied by Cambridge Isotope Laboratories, Andover, MA, USA. Stock solutions of all standards (1000 mg L^−1^) were prepared from which working standard solutions (10 mg L^−1^) were made in methanol and then stored in a freezer at −18 °C. Organic solvents of high-performance liquid chromatography (HPLC) grade including acetonitrile, methanol, 2-propanol, and formic acid were purchased from Sigma-Aldrich, Sydney, Australia. Three different photocatalysts were used, including Degussa P25 TiO_2_,(Degussa AG, Frankfurt, Germany), Aldrich TiO_2_ (Sigma Aldrich, Sydney, Australia), and Hombikat UV100, (Sachtleben Chemie GmBH, Duisburg, Germany), and their characteristics are summarized in Table 1. Ultra-pure water was provided from a Milli-Q water system. The Oasis^®^ HLB solid-phase extraction (SPE) cartridges (6 mL/200 mg) were obtained from Waters Corporation, Wilmslow, UK. Natural aquatic colloids (representing dissolved organic matter or DOM) were isolated from a range of water samples by using cross-flow ultrafiltration [39], and subsequently characterized using solid-state ^13^C nuclear magnetic resonance in the chemical shift region (110–160 ppm) on Avance DMX400 (Bruker, Billerica, MA, USA) for aromatic carbon content.

In addition, sewage effluent samples were taken from a STW in Sydney, NSW, Australia. The effluent quality was measured for pH (6.8–7.9), dissolved oxygen (5.4–7.1 mg L^−1^), temperature (8.6–11.8 °C), COD (76–105 mg L^−1^), and BOD_5_ (11–27 mg L^−1^). For the photocatalysis experiments, effluent samples were collected in pre-cleaned amber glass bottles (2.5 L) and spiked with sodium azide (final conc. = 0.02 M) to minimize potential biodegradation. On return to the laboratory, the samples were immediately filtered using pre-combusted Whatman (Banbury, UK) GF/F filter paper (0.7 μm) to remove particulate matter, and spiked with 100 ng each of internal standards. Then, the samples were ready for solid-phase extraction (SPE) to measure the concentrations of the pharmaceuticals.

### 2.2. Photocatalysis

Two photo-reactors designed by Heraeus Noblelight (Hanau, Germany) were used in this study, equipped with TQ 150 (medium-pressure Hg-vapor lamp, 150 W) and TNN 15–32 (low-pressure Hg-vapor lamp, 15 W), respectively. The TQ 150 emitted a continuous wavelength (238–579 nm), and due to the filtering by the glass jacket, its effective wavelength was in the near UV to visible range. The TNN 15–32 was housed in a quartz jacket, with intense UV irradiation at 253 nm.

The working water samples were prepared by spiking stock solutions (10 mg L^−1^) to ultrapure water (0.4 L and 0.7 L for TQ150 and TNN 15–32, respectively) to obtain an initial concentration of 18–1000 ng L^−1^. A magnetic stirrer was used to stir the solutions (for 30 min) before TiO_2_ was added at different concentrations. Control experiments conducted in the presence of TiO_2_ but absence of light showed a very limited loss of the three compounds (<2%). The prepared solutions were then transferred to the photo-reactors to start the photocatalysis experiments. For extraction and analysis, samples (e.g., 10 mL) were taken from the reactor vessel at different time intervals. The photocatalytic experiments were performed under a variety of conditions such as different solution pH, different concentrations of pharmaceuticals or TiO_2_ photocatalyst, and the presence of precursors and inhibitors of ^•^OH, to identify the mechanism.

For sewage effluent samples, the photocatalytic experiments were conducted for both the original sewage effluents and effluents spiked with 50 ng L^−1^ of the target compounds. Once completed, the effluent samples were processed using SPE, in the same way as for water samples.

### 2.3. Extraction and Analytical Procedures

The water and sewage effluent samples were extracted using SPE, following a method developed previously [40]. Briefly, the Oasis HLB (Waters) SPE cartridges were conditioned with 10 mL of methanol, followed by ultrapure water (3 × 10 mL) at a rate of 1–2 mL min^−1^. Then, water samples were extracted using SPE at a flow rate of 5–10 mL min^−1^. Afterwards, the cartridges were dried for 30 min under vacuum, with the analytes being eluted with 10 mL of methanol which was reduced to 0.1 mL under a gentle N_2_ flow. 

HPLC-electrospray ionization-tandem mass spectrometry (HPLC-ESI-MS/MS) was used for the analysis of pharmaceuticals, following a method developed by Zhou et al. [40]. Briefly, sample extracts (10 µL) were injected into a Waters 2695 HPLC separations module (Waters Corporation, Milford, MA, USA), for separation on a Waters Symmetry C_18_ column (2.1 × 100 mm, particle size 3.5 µm). The mobile phase comprised eluent A (with 0.1% formic acid in ultrapure water), eluent B (acetonitrile), and eluent C (methanol). At a flow rate of 0.2 mL min^−1^, the elution started with eluent B (10%), followed by a 25 min gradient to eluent B (80%) and a 3 min gradient to eluent B (100%), and then changed to eluent C (100%) within 8 min, held for 10 min and then reverting to the initial conditions. The MS/MS analyses were performed by using a Micromass Quattro triple-quadrupole mass spectrometer (Waters, Wilmslow, UK) in the positive ion mode. The ESI source block and desolvation temperature was 100 and 300 °C, respectively; the capillary and cone voltage was 3.0 kV and 30 V, respectively; the argon collision gas was at 3.6 × 10^−3^ mbar; the flow rate for the cone nitrogen gas and desolvation gas was 25 and 550 L h^−1^. The collision energy, cone voltage, and transitions chosen for the multiple reaction monitoring experiment were optimized.

## 3. Results and Discussion

### 3.1. Kinetics of Pharmaceutical Photocatalysis in Water

**Figure 1:**
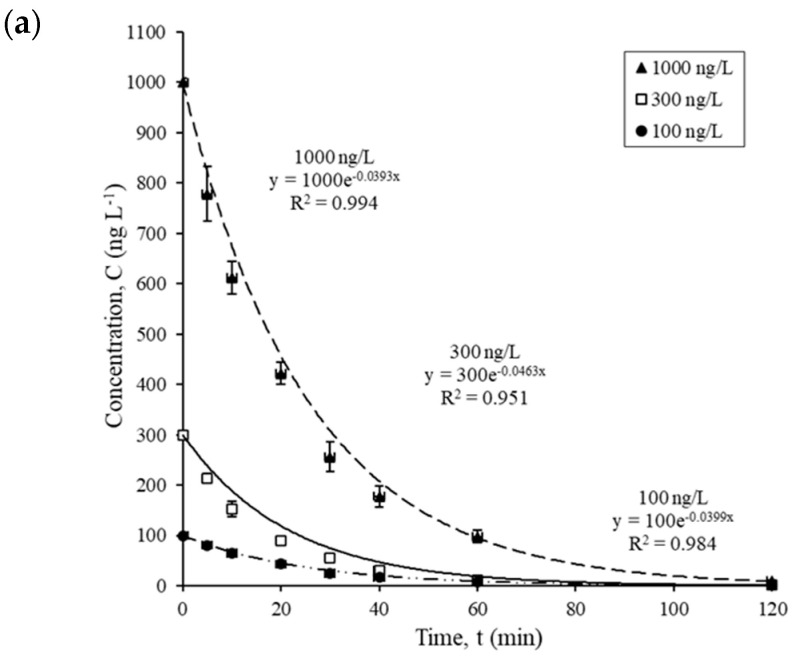

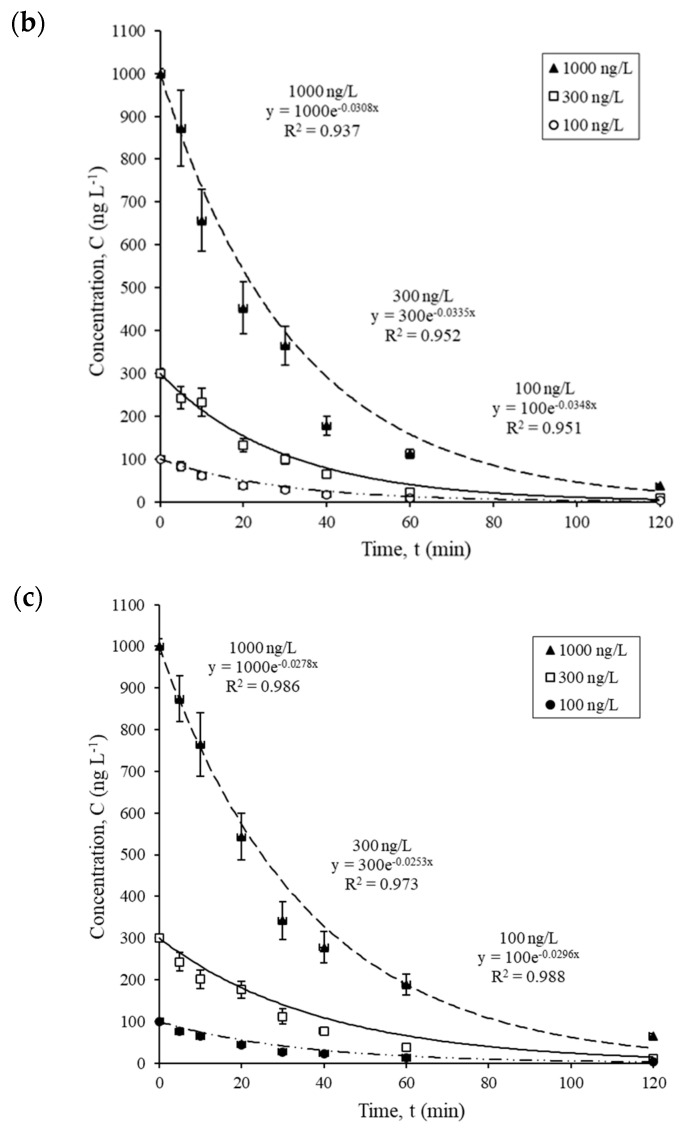
Photocatalysis kinetics of three different concentrations of (**a**) propranolol, (**b**) mebeverine, and (**c**) carbamazepine using Degussa P25 TiO_2_ (150 mg L^−1^) under irradiation with TQ 150.

The kinetic experiments were performed using a polychromatic lamp (TQ 150, Heraeus Noblelight, Hanau, Germany) with a continuous wavelength of 238–579 nm, under different initial pharmaceutical concentrations although the photocatalyst dosage was kept at 150 mg L^−1^. As shown in Figure 1a–c, the concentration of propranolol, mebeverine, and carbamazepine decreased rapidly with time. After 30 min of irradiation, approximately 76.7% of propranolol, 67.3% of mebeverine, and 67.2% of carbamazepine were degraded. At the end of 2 h irradiation, 99.7% of propranolol, mebeverine, and carbamazepine were degraded, suggesting a rapid degradation process.

**Figure 2:**
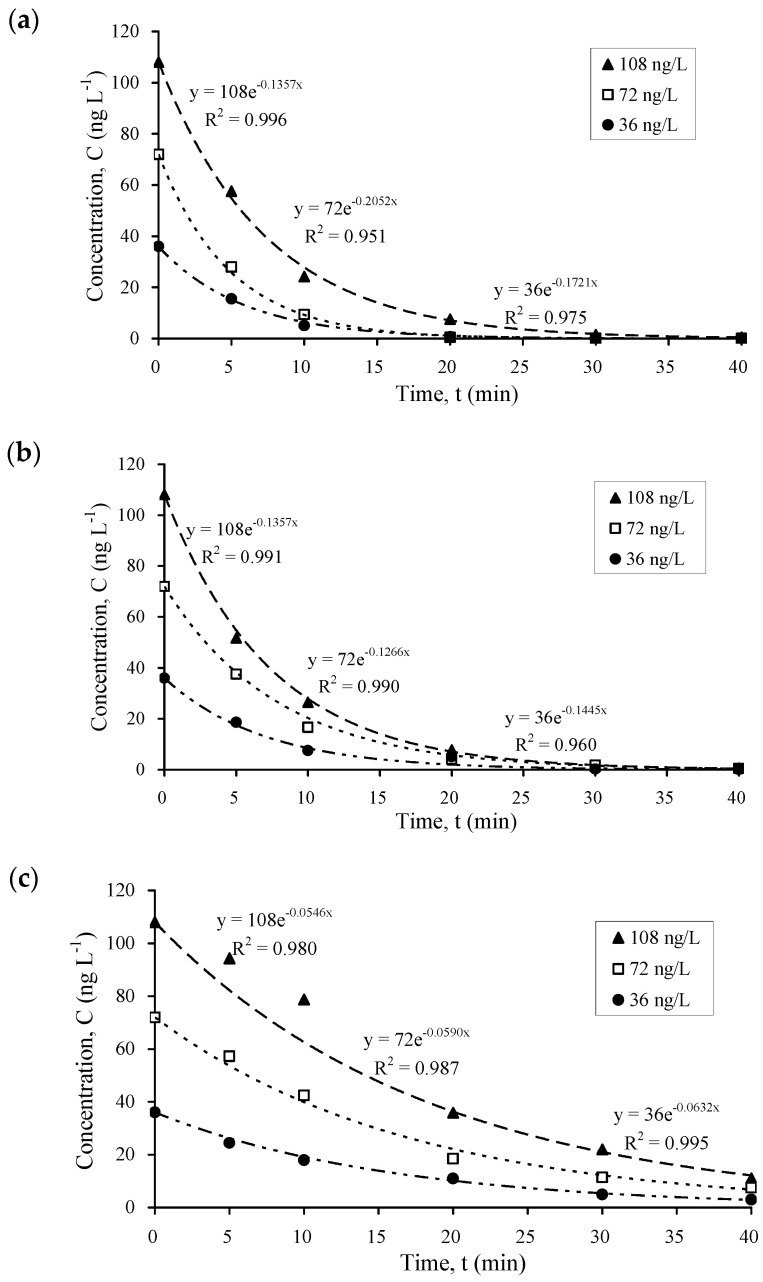
Kinetics of photocatalysis of (**a**) propranolol, (**b**) mebeverine, and (**c**) carbamazepine using Degussa P25 TiO_2_ (150 mg L^−1^) under irradiation with TNN 15–32. Initial pharmaceutical concentration = 36, 72, and 108 ng L^−1^. Error bars ≤ 18%.

When a monochromatic lamp (TNN 15–32) emitting at a single wavelength of 253 nm was used, the results (Figure 2) showed an even more rapid degradation than that using a continuous wavelength (TQ 150). The disappearance of 99.3% of propranolol, 98.5% of mebeverine, and 83.2% of carbamazepine was achieved after 30 min of irradiation. The photodegradation kinetics were also relatively independent of the initial pharmaceutical concentration (from 36–108 ng/L), which are similar to the results under the continuous wavelength irradiation (Figure 1). The results can be explained by the light absorption in the UV region of these three compounds with the absorbance peaks at 215 nm for propranolol, 221 nm and 263 nm for mebeverine, and 211 nm and 285 nm for carbamazepine, which are very close to the emission wavelength of 253 nm.

To investigate the apparent rate constant of pharmaceutical degradation, a first-order kinetics model was applied as follows [41]:

(2) $$\ln\left(\frac{C}{C_{0}}\right)=-kt$$

where *C*_0_ and *C* are the concentrations at time zero and time t (min), and *k* is the first-order photodegradation rate constant (min^−1^). The results indicated that the UV photocatalytic kinetics of the three pharmaceutical compounds in aqueous solution were in accordance with the first-order law. 

The kinetics data are consistent with those of Martínez et al. [42] who observed spectral changes of carbamazepine upon irradiation with UV at 254 nm, and that 95% of carbamazepine was transformed after 30 min of irradiation. In addition, Martínez et al. [43] found that 90% of initial diclofenac was degraded using photocatalysis within 30 min. Martínez et al. [42,43] suggested that the photocatalysis of carbamazepine and diclofenac was more effective under UV than that under near UV-visible irradiation. Considering Figure 2c, the calculated rate constants of photocatalysis under UV irradiation for carbamazepine (0.055–0.063 min^−1^) were smaller than 0.1521 min^−1^ that was obtained by Martínez et al. [42], but higher than 0.0029 min^−1^ reported by Haroune et al. [5]. Such a difference could be partly related to the high pharmaceutical concentrations being used in other studies, e.g., 8 mg L^−1^ [42], which are significantly higher than those concentrations being detected in sewage effluent and river waters often at ng L^−1^ to µg L^−1^ range. The findings therefore demonstrate the importance of conducting photocatalytic experiments at environmentally relevant concentrations, to ensure that the findings can be used for field application, plant design, and scale-up in STWs.

### 3.2. Performance of Different Photocatalysts at Different Dosages

**Figure 3:**
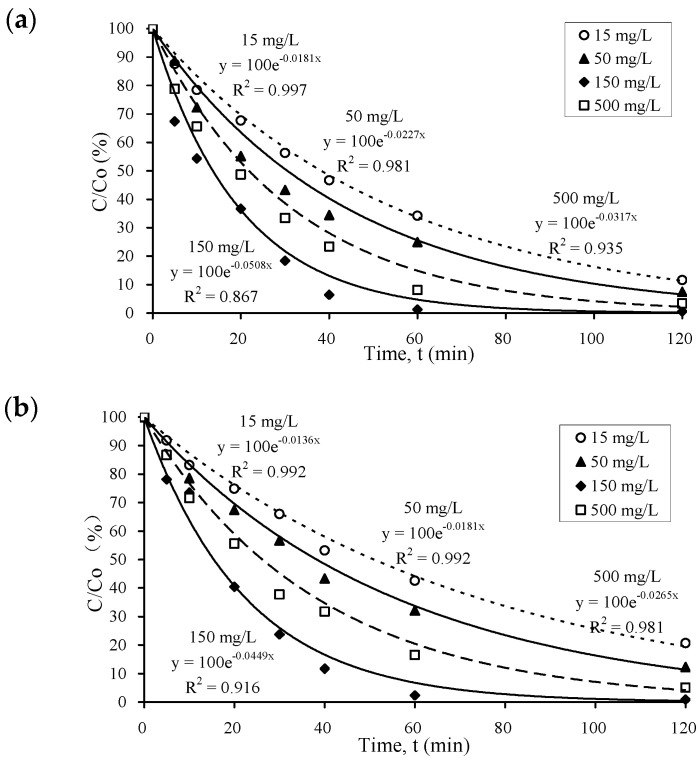

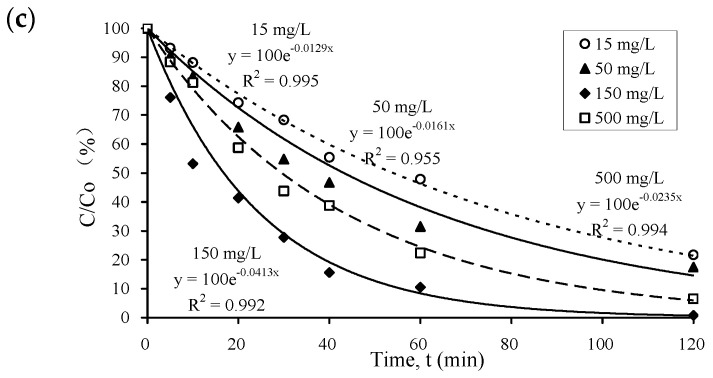
The photocatalysis of (**a**) propranolol, (**b**) mebeverine, and (**c**) carbamazepine at different dosages of Aldrich TiO_2_ under irradiation using TNN 15–32 (initial pharmaceutical concentration = 100 ng L^−1^). Error bars ≤ 15%.

To fully understand the mechanism of photocatalysis, three different photocatalysts were tested under irradiation using TNN 15–32. Like Degussa P25, the TiO_2_ photocatalyst from Aldrich also displayed potential in the degradation of pharmaceuticals (Figure 3). The photodegradation kinetics also followed the first-order model. In addition, the rate of photocatalysis was highly dependent on the dosage of the photocatalyst, improving with increasing the photocatalyst dosage up to 150 mg L^−1^. A further increase in the photocatalyst dosage to 500 mg L^−1^ was shown to reduce the rate of photocatalysis. The increase in rate constant with catalyst dosage up to 150 mg L^−1^ can be explained by the positive relation between the photocatalyst dosage and the abundance of available active sites on TiO_2_. However, at the highest photocatalyst dosage (500 mg L^−1^), the total available active sites on TiO_2_ may be reduced as a result of particle interactions such as coagulation forming visible aggregates. For instance, it has been reported by So et al. [44] that the agglomeration and sedimentation of TiO_2_ particles occurred at 2000 mg L^−1^. In addition, light penetration through TiO_2_ suspension can be adversely affected by the relatively high catalyst dosage, due to the scattering of incident light [45], causing a reduction in the efficiency of TiO_2_.

As shown in Table 2, the performance of photocatalysis was dependent on several operating parameters, which became more efficient using irradiation under UV light than under UV-visible light. Secondly, the photodegradation was most effective with Degussa P25, followed by Hombikat 100 TiO_2_, and finally by Aldrich TiO_2_, suggesting the critical role of the crystal structure on the photocatalytic activity of TiO_2_. Notably, Degussa P25 TiO_2_ consists of anatase and rutile phases and is generally known as a benchmark photocatalyst [25]. Thirdly, the photocatalysis was most efficient when the photocatalyst dosage was 150 mg L^−1^. When studying diclofenac photocatalysis, Achilleos et al. [34] and Martínez et al. [42] observed that a pure anatase photocatalyst exhibited a greater photocatalytic activity than a pure rutile photocatalyst. Such a difference in photocatalytic activity between anatase and rutile TiO_2_ is likely to be due to some factors such as the more positive position of the conduction band for the rutile phase, the lower recombination rate of e^−^/h^+^ pairs in the anatase phase, and the higher capacity of the anatase phase for the adsorption of oxygen (owing to the higher density of superficial hydroxyl groups) [46]. In addition, it should be noted that Hombikat UV100 had a larger specific surface area than Aldrich TiO_2_ (Table 1), hence favoring electron transfer amongst electrons, holes, and the reactants [47], and improving the efficiency of Hombikat UV100 compared with Aldrich TiO_2_.

Notably, Martínez et al. [42,43] observed an optimum photocatalyst dosage of 0.5 g L^−1^ and 1.0 g L^−1^ for the photodegradation of carbamazepine and diclofenac, respectively, using Degussa P25. By increasing the dosage of the photocatalyst, the number of active sites for the absorption of light and subsequent photodegradation will be enhanced. On the other hand, the amount of light dispersed by the photocatalyst particles will also be increased, making some of the photocatalyst particles unavailable for the generation of h^+^/e^−^ pairs. Furthermore, some of the originally activated TiO_2_ may also be deactivated through collision with ground-state molecules [48]. As a result, an optimum concentration of 150 mg L^−1^ P25 TiO_2_ was recommended for the subsequent and future photocatalytic experiments.

### 3.3. Effect of DOM on the Photocatalytic Degradation Rate

In this work, the effect of water and wastewater properties such as pH and DOM on the pharmaceutical photodegradation was carefully examined. When the photocatalysis experiments were conducted at different pH values between 6 and 9, no significant effect was observed on the reaction kinetics. Similarly, when using TiO_2_ as the photocatalyst, the percentage phototransformation of carbamazepine was not affected between pH 3.0 and 6.4 [5]. However, the researchers reported that carbamazepine photo-oxidation was significantly inhibited at a very high pH of 11.

**Figure 4:**
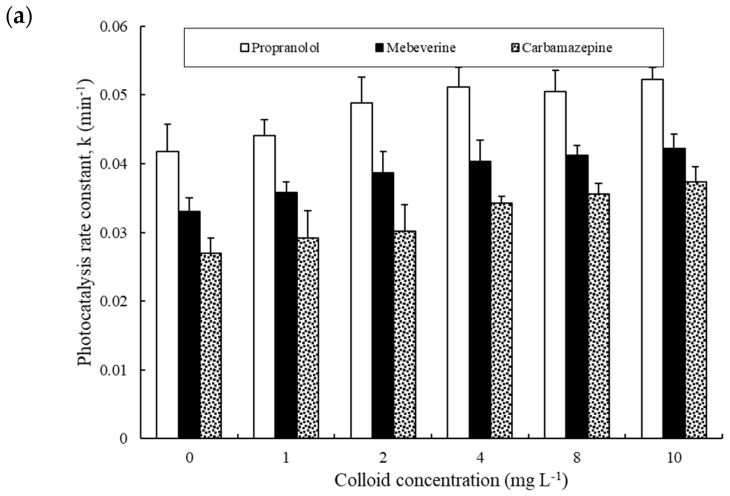

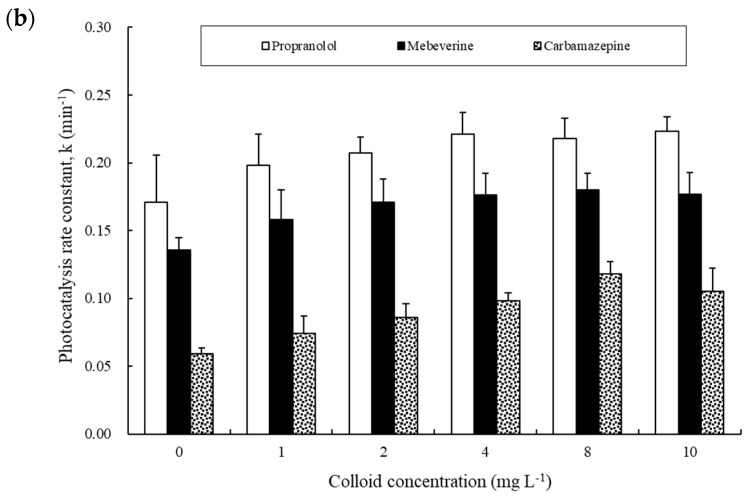
Effect of aquatic colloids on the photocatalysis of propranolol, mebeverine, and carbamazepine (initial concentration = 500 ng L^−1^) under irradiation using (**a**) TQ 150 and (**b**) TNN 15–32 in the presence of 150 mg L^−1^ of Degussa P25.

In comparison, the presence of aquatic colloids in water enhanced the photodegradation rate constant when being irradiated using lamp TQ 150 (Figure 4a). When a different lamp (TNN 15–32) was used, a similar enhancement trend was observed (Figure 4b). These results confirmed the important role of aquatic colloids as chromophores in absorbing photons, which is likely followed by the production of radicals, subsequently inducing the indirect photolysis of the pharmaceuticals [5]. In studying the DOM effect, the large majority of research was conducted using humic substances which were obtained by chemical extraction methods, during which harsh chemicals such as NaOH and HCl solutions may have altered the structure of natural organic matter [39]. Here, natural aquatic colloids were isolated using a physical method (cross-flow ultrafiltration) which is more representative of natural DOM than chemically extracted humic material. In addition, various studies show that aquatic colloids are important repositories of emerging pollutants such as EDCs and pharmaceuticals in the aquatic environment [6,39].

The effects of DOM on the photocatalysis of organic contaminants have been widely reported, and can include both positive and negative influences. This is likely due to the counter forces from DOM, which can absorb light or react with radicals causing a reduction in photodegradation, or which becomes a photosensitizer, thus enhancing photodegradation [5,49]. This is further compounded by the high diversity and chemical complexity of DOM molecules, which are often poorly characterized. In studying the photodegradation of three antiviral drugs, Zhou et al. [36] reported that different DOMs promoted their photodegradation, and the promotion effect of seawater DOM was weaker than that of freshwater DOM.

**Figure 5:**
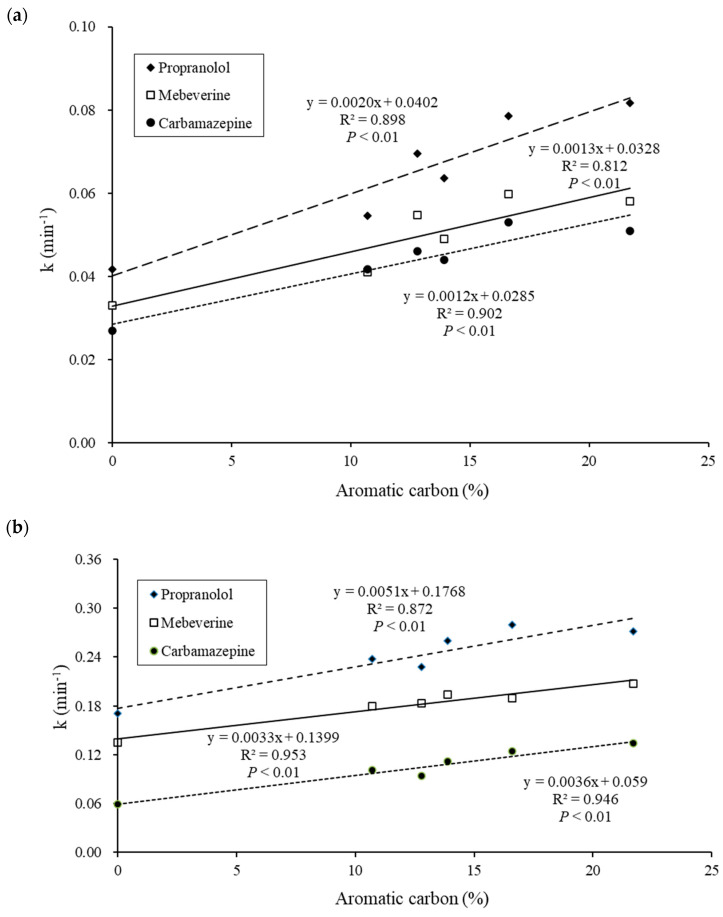
Relationship between the photocatalysis rate constant and the aromatic carbon content of aquatic colloids during irradiation using 150 mg L^−1^ of (**a**) TQ 150 and (**b**) TNN 15–32 (initial pharmaceutical concentration = 100 ng L^−1^).

To further understand the mechanism of the colloidal DOM effect on photocatalysis, different aquatic colloids that demonstrated a photosensitizing effect were analyzed for elemental composition and the contents of functional groups such as aromatic and aliphatic carbon content. Through statistical analysis, a positive relationship was observed between colloidal aromatic carbon content and the rate constant of pharmaceutical photocatalysis (Figure 5). Chin et al. [50] observed significant bisphenol A transformation in the presence of humic substances, and their effect was loosely correlated to the carboxyl carbon content. In a study of estrone photodegradation, Caupos et al. [51] found that its kinetics were enhanced by DOM, with the effect being more significant the higher the fluorescence efficiency of DOM. In addition, Leech et al. [52] observed that the photodegradation of 17β-estradiol was enhanced in the presence of aquatic humic acids. However, no significant effect of humic acid was observed during the photocatalytic degradation of carbamazepine and three derivatives [5]. In another study by Mohapatra et al. [35], humic acid reduced the photodegradation of diclofenac and sulfamethoxazole but increased the photodegradation of acetaminophen, carbamazepine, and gemfibrozil. They suggested the direct photodegradation of diclofenac and sulfamethoxazole, and the indirect photodegradation of acetaminophen, gemfibrozil, and carbamazepine. In a study of the DOM effect on the photodegradation of antiviral drugs, the photodegradation of acyclovir was mainly promoted by ^3^DOM*, and the photodegradation of lamivudine was accelerated by a combination of ^3^DOM*, ^1^O_2,_ and ^•^OH radicals [36]. Furthermore, in studying the photodegradation of 17β-estradiol in water, Zhou et al. [10] reported that a higher mediation efficiency of dissolved black carbon than humic substances was caused by the higher aromatic content and smaller molecular sizes.

### 3.4. Probing of Radicals Involved in Pharmaceutical Photocatalysis

**Figure 6:**
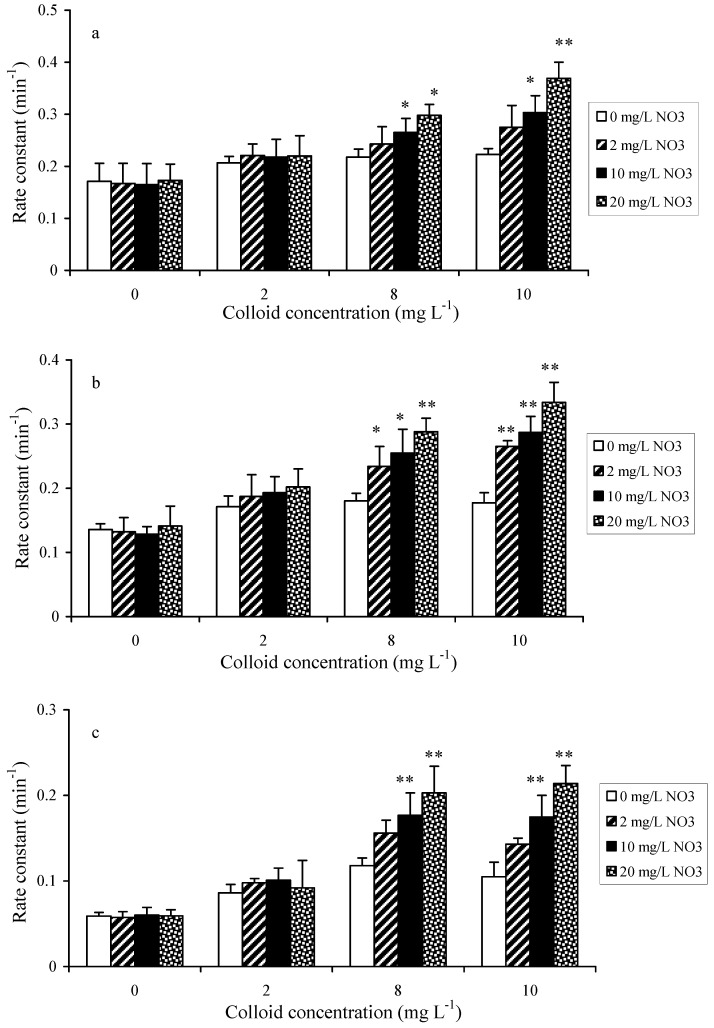
Effect of nitrate as a photosensitizer on the photocatalysis rate constant for (**a**) propranolol, (**b**) mebeverine, and (**c**) carbamazepine. Symbols “*” and “**” indicate *p* < 0.05 and *p* < 0.01, respectively (initial pharmaceutical concentration = 100 ng L^−1^, light = TNN 15–32).

It is well known that sunlight-irradiated DOM can result in the formation of ROS like hydroxyl radicals (^•^OH), singlet oxygen (^1^O_2_), and ^3^DOM*, thereby improving the photodecomposition of organic pollutants [53]. The positive effect of aquatic colloids on pharmaceuticals photocatalysis can be interpreted by the so-called photosensitizing effect, in particular the production of ROS. To examine which types of radicals are particularly important in the degradation process, a range of experiments were conducted to examine the importance of hydroxyl radicals and their scavengers. First, the effect of NaNO_3_ was tested, as it is widely present in wastewater and natural water. In addition, during water treatment, the irradiation of nitrate by UV can generate radicals such as ^•^OH [54]. A range of nitrate concentrations (up to 20 mg L^−1^) was added to the solutions of pharmaceuticals and colloid mixtures to assess the nitrate impact on the photodegradation rate constant (Figure 6). At low concentrations of aquatic colloids (≤2 mg L^−1^), the presence of nitrate had no appreciable effect on the photodegradation rate constants of the pharmaceuticals. When aquatic colloids were present at relatively high concentrations (8 and 10 mg L^−1^), the presence of nitrate was shown to enhance the photodegradation rate constants, and the effect became more significant with increasing the concentration of nitrate. The findings would suggest some sort of synergistic effect between the aquatic colloids and nitrate to promote the production of ROS such as hydroxyl radicals when both are present at relatively high concentrations. Based on this set of experiments, the highest rate constants for the photodegradation of propranolol, mebeverine, and carbamazepine were found to be 0.369 min^−1^, 0.334 min^−1^, and 0.214 min^−1^, which were equal to a half-life of 1.9 min, 2.1 min, and 3.2 min, respectively.

Zhan et al. [55] also observed a complex relationship between nitrate, humic substances, and bisphenol A photodegradation, which was dependent on their respective concentrations. Hence, the results suggest that the photocatalysis of the three pharmaceuticals is likely to be facilitated by the presence of hydroxyl radicals.

**Figure 7:**
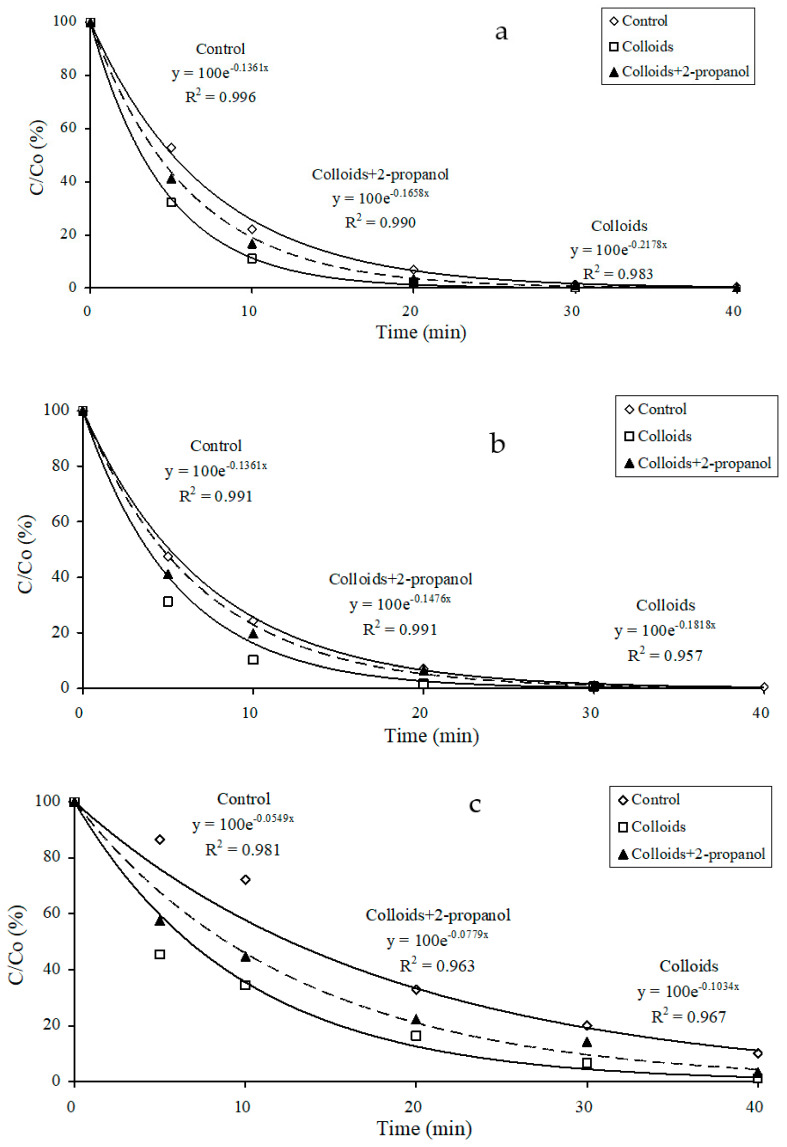
Effect of 2-propanol (0.1 M) as an ^•^OH scavenger on the photocatalysis of (**a**) propranolol, (**b**) mebeverine, and (**c**) carbamazepine when irradiated using TNN 15–32 (initial pharmaceutical concentration = 100 ng L^−1^).

To further differentiate between hydroxyl-radical- and non-hydroxyl radical-mediated photolysis pathways, the influence of radical scavenging was examined. The results (Figure 7) show that 2-propanol (0.1 M) was found to inhibit the kinetics of photocatalysis of propranolol, mebeverine, and carbamazepine. However, at the concentration of 0.01 M, the effect of 2-propanol was insignificant. These results confirmed that the photodegradation of these pharmaceutical compounds could be likely through indirect photolysis by hydroxyl radicals. Similarly, Zhan et al. [55] observed that the photosensitized degradation of bisphenol A was significantly inhibited by 0.14 M of 2-propanol. During the study of bisphenol A photodegradation, Chin et al. [50] found that the addition of methanol slowed its indirect photolysis as a result of the ^•^OH scavenge effect. Similarly, Leech et al. [52] observed a significant reduction in the photodegradation rate of 17β-estradiol on the addition of 2-propanol (2% *v*/*v*), and Zhou et al. [36] reported the inhibition of acyclovir and lamivudine photodegradation from the addition of isopropanol.

### 3.5. Photocatalytic Removal of Pharmaceuticals in Sewage Effluent

Further experiments were conducted by examining the potential destruction of the pharmaceutical compounds in sewage effluents under the influence of photocatalysis. The results showed that the disappearance of the three compounds from filtered sewage effluent samples (4.3 ng L^−1^ propranolol, 6.6 ng L^−1^ mebeverine, and 2.5 ng L^−1^ carbamazepine) was rapid in the presence of 150 mg L^−1^ of Degussa P25 when irradiated using TNN 15–32, and within 20 min, none of them was detected. A further set of experiments involving spiking 50 ng L^−1^ of the three compounds in sewage effluent demonstrated that the rate constants of photodegradation were 0.28 ± 0.04 min^−1^, 0.21 ± 0.04 min^−1^, and 0.15 ± 0.03 min^−1^, respectively, which were close to those obtained in water samples. The findings are interesting as sewage effluent is far more complex than natural waters containing many trace contaminants, and such a high performance with undiminished kinetics indicates the great potential of photocatalysis in rapidly degrading pharmaceuticals in wastewater. In addition, the extraction and LC-MS analysis of sewage effluent treated using photocatalysis showed no intermediate compounds derived from the three target pharmaceuticals, suggesting their potential mineralization at environmentally relevant concentrations.

## 4. Conclusions

The photocatalytic degradation of propranolol, mebeverine, and carbamazepine was studied under UV and UV-visible irradiation using three different TiO_2_ nanostructures. The results showed that the photocatalysis of pharmaceuticals was influenced by several parameters, in particular, the source of light and the type of photocatalysts. In essence, the photocatalysis rate constant was more rapid under UV light than under UV-visible light, and more effective by using Degussa P25 TiO_2_ than that using Hombikat UV100 or Aldrich TiO_2_. Notably, the kinetics of the photocatalytic degradation of propranolol, mebeverine, and carbamazepine followed the first-order law with an estimated half-life of 4.0–8.8 min, 5.1–11.1 min, and 11.8–23.3 min, respectively, under UV irradiation with P25 as the photocatalyst; and the presence of aquatic colloids increased the photodegradation of the pharmaceuticals. The photodegradation rate constant was further increased by the addition of nitrate, showing a potential synergistic effect with DOM. Notably, the addition of 2-propanol (0.1 M) reduced the rate constant of photocatalysis, demonstrating the important role of ^•^OH in the photocatalytic degradation of the pharmaceuticals. The optimized photocatalytic conditions were subsequently used in treating STW effluents, with performance (e.g., kinetics) comparable to that observed in water. With increasing concern over pharmaceuticals and other emerging contaminants in aquatic systems, photocatalysis showed its potential as an effective, rapid, and green method of removing such contaminants, thus protecting aquatic ecosystems and public health.

## Figures and Tables

**Table 1:** Properties of different TiO_2_ nanostructures used in photocatalytic experiments.

Photocatalyst	Degussa P25	Aldrich	Hombikat UV100
Manufacturer	Degussa AG	Aldrich	Sachtleben Chemie GmBH
Density (g mL^−1^) at 20 °C	3.8	4.17	3.9
Average diameter of primary particles (nm)	21	72	5
BET surface area (m^2^ g^−1^)	50	152	270
Anatase by XRD (%)	70	5	100
Rutile by XRD (%)	30	95	0

**Table 2:** A summary of the rate constants from the photocatalytic degradation of the pharmaceutical compounds under different operating conditions.

Photocatalyst	Photocatalyst Dose (mg L^−1^)	Reactor	Propranolol		Mebeverine		Carbamazepine	
			k (min^−1^)	t½ (min)	k (min^−1^)	t½ (min)	k (min^−1^)	t½ (min)
Degussa P25	50	TQ 150	0.032 ± 0.005	21.7	0.028 ± 0.006	24.8	0.019 ± 0.004	36.5
	150		0.042 ± 0.004	16.5	0.033 ± 0.002	21.0	0.027 ± 0.002	25.7
	15	TNN 15–32	0.079 ± 0.006	8.8	0.062 ± 0.006	11.2	0.030 ± 0.006	23.1
	50		0.102 ± 0.014	6.8	0.089 ± 0.016	7.8	0.040 ± 0.008	17.3
	150		0.171 ± 0.035	4.1	0.136 ± 0.009	5.1	0.059 ± 0.004	11.7
Aldrich	15	TNN 15–32	0.018 ± 0.002	38.5	0.014 ± 0.003	49.5	0.013 ± 0.002	53.3
	50		0.023 ± 0.002	30.1	0.018 ± 0.003	38.5	0.016 ± 0.001	43.3
	150		0.051 ± 0.003	13.6	0.045 ± 0.005	15.4	0.041 ± 0.003	16.9
	500		0.032 ± 0.004	21.7	0.027 ± 0.002	25.7	0.024 ± 0.002	28.9
Hombikat UV100	15	TNN 15–32	0.027 ± 0.003	25.7	0.020 ± 0.001	34.7	0.018 ± 0.002	38.5
	50		0.030 ± 0.004	23.1	0.028 ± 0.003	24.8	0.022 ± 0.003	31.5
	150		0.058 ± 0.004	11.9	0.048 ± 0.004	14.4	0.042 ± 0.004	16.5
	500		0.039 ± 0.002	17.8	0.032 ± 0.004	21.7	0.031 ± 0.005	22.4

## Data Availability

The datasets used and/or analyzed during the current study are available from the corresponding author on reasonable request.
